# Traumatic Erysipelas Aborted

**Published:** 1890-11

**Authors:** D. Du Pre

**Affiliations:** Dallas, Texas


For Daniel's Texas Medical Journal.

### TRAUMATIC ERYSIPELAS ABORTED.

BY D. DU PRE, M. D., DALLAS, TEXAS.

MR. R., a mechanic working on the new county bridge connecting Oak Cliff with Dallas, was standing at the foot of the piling, when a block of green cypress timber 10x10 and about 24 inches in length, was detached by being sawn off about 20 feet above him, and in its decent struck his head on the line of the coronal sutures, making an incision five inches in length, turning back the scalp with a portion of the periosteum, and denuding about one half of the occiput. He was insensible only a few minutes. His escape from instantaneous death may be attributable to the sounding of alarm causing a sudden looking up, so that the missile struck his head obliquely. There was only a slight abrasion of right shoulder and no real injury to the bone. The periosteum was restored as far as possible to its position, and would have been stitched, had animal suture been at command. Drainage at most dependant angle of wound was established. Ten sutures were required to close the wound. The accident occurred during the forenoon of the 7th inst.

About noon of the 8th he had a rigor, followed with fever and severe cephalalgia. His bowels were opened with C. C. pills during the first twelve hours. The early morbid symptoms inferred to shock and traumatic irritation, and was treated accordingly. The day following there was a return of the morbid conditions of the preceding day, but of increased severity. Before night-fall there were developed the flush and eruption of erysipelas, delirium and other constitutional symptoms alarming and rampant. The œdema and eruption involved neck, face and scalp.

I at once entered upon the practice of my life, to-wit: the prompt and vigorous application of remedies in all like cases of malignant disease.

I ordered sodii salicyl. gr. x., potassii nitrat. gr. v., every two hours until the pyrexia was under control; and phenacetin gr. v. and codeine gr. ss. every two hours to doubly insure analgesic and antithermic effect. The conditions were promptly met as sought. The next day there was no return of the paroxysms of the two previous days.

I felt in the constitutional remedies used of the ℞ there could be no safer or more effective antipyretics, antiseptics and germicides when important to immediate *saturation* of the system for remedial ends. As to local applications, I selected solution of potass. permang. gr. v. to f. ℥i. of aqua pura applied three or four times daily, and the parts protected with absorbent cotton saturated with acid boric. in solution with water and glycerine.

The result: In twenty-four hours there was the arrest of all local inflammatory action, and almost entire subsidence of œdema. This is the 7th day, and there is neither local nor constitutional disturbance.

The sutures were removed at the end of sixty hours. No suppuration of line of incision and almost union by first intention and a like union of two-thirds of scalp. The surface where the periosteum was detached discharged bloody serum for five days, is now becoming more colorless, less abundant and with globules, sparcely distributed, of pus. The sinus is daily syringed out with hydras, canaden, alb. and acid boric. in solution with glycerine and water. For the last two dressings, I injected f. ʒi. of iodoform in solution with sulph. ether ʒi. to f. ℥i. The favorable change in discharge I attribute to the latter. Its burning sensation only lasts a few moments.

This mode of treatment is a *departure* from that usual in such cases. As traumatic erysipelas is looked upon as a most dangerous complication in grave injuries of the class under consideration, the prognosis generally bad—that of chronicity or death, I did not feel justifiable in adopting the usual line of treatment, which has generally fallen short of the prompt arrest of this disease, to which we should direct our efforts. I had no time to lose. The prodrome in this case, and character of eruption, febrile stage and other systemic and local disturbances will readily differentiate it.

I am confident the early curative results in this case were referable to treatment detailed, and do not hesitate to emphasize my confidence in its efficacy for future application, notwithstanding it is my first venture.

Permanganate of potash has rarely disappointed me as a local remedy in the various forms of erysipelas. I have felt impressed to call the attention of the profession to this case.

Seen last on the 15th; wound suppurating.

---

To Remove Warts.—℞. Meg. hyd. nit., gr. c. Arsen. alba, gr. v—x. M. ft. meg. Sig.: Spread on linen and apply over wart. The growth will soften and gradually disappear without pain.—*Ex.*

---

Aural Furuncle.—℞. Menthal, gr. xv. Ol. amygdolor Dulc. ʒ v. M. Sig.: Introduce twice daily on cotton.—*Ex.*

